# Concurrent ibrutinib plus venetoclax in relapsed/refractory mantle cell lymphoma: the safety run-in of the phase 3 SYMPATICO study

**DOI:** 10.1186/s13045-021-01188-x

**Published:** 2021-10-30

**Authors:** Michael Wang, Radhakrishnan Ramchandren, Robert Chen, Lionel Karlin, Geoffrey Chong, Wojciech Jurczak, Ka Lung Wu, Mark Bishton, Graham P. Collins, Paul Eliadis, Frédéric Peyrade, Yihua Lee, Karl Eckert, Jutta K. Neuenburg, Constantine S. Tam

**Affiliations:** 1 Department of Lymphoma and Myeloma, University of Texas MD Anderson Cancer Center, 1515 Holcombe Blvd #368, Houston, TX 77030 USA; 2 University of Tennessee, Knoxville, TN USA; 3 City of Hope National Medical Center, Duarte, CA USA; 4 Centre Hospitalier Lyon Sud, Lyon, France; 5 Olivia Newton-John Cancer Centre, Heidelberg, VIC Australia; 6 Sklodowska Curie National Research Institute of Oncology, Kraków, Poland; 7 Ziekenhuis Netwerk Antwerpen, Antwerp, Belgium; 8 Nottinghamshire University Hospitals, Nottingham, UK; 9 NIHR Oxford Biomedical Research Centre, Churchill Hospital, Oxford, UK; 10 Icon Cancer Centre, South Brisbane, QLD Australia; 11 Centre Antoine Lacassagne, Nice Cedex 2, France; 12 Pharmacyclics LLC, an AbbVie Company, Sunnyvale, CA USA; 13 Peter MacCallum Cancer Centre, Royal Melbourne Hospital, St Vincent’s Hospital, University of Melbourne, Melbourne, VIC Australia

**Keywords:** Hematological cancers/lymphomas, Small molecule agents/kinase inhibitors, Ibrutinib, Venetoclax, Safety

## Abstract

Ibrutinib plus venetoclax, given with an ibrutinib lead-in, has shown encouraging clinical activity in early phase studies in mantle cell lymphoma (MCL). The ongoing phase 3 SYMPATICO study evaluates the safety and efficacy of concurrently administered, once-daily, all-oral ibrutinib plus venetoclax in patients with relapsed/refractory MCL. A safety run-in (SRI) cohort was conducted to inform whether an ibrutinib lead-in should be implemented for the randomized portion. Patients received concurrent ibrutinib 560 mg continuously plus venetoclax in a 5-week ramp-up to venetoclax 400 mg for up to 2 years. The primary endpoint was occurrence of tumor lysis syndrome (TLS) and dose-limiting toxicities (DLTs). The SRI cohort enrolled 21 patients; six and 15 were in low- or increased-risk categories for TLS, respectively. During the 5-week venetoclax ramp-up, three patients had DLTs, and one patient at increased risk for TLS had a laboratory TLS; no additional TLS events occurred during follow-up. With a median follow-up of 31 months, the overall response rate was 81% (17/21); 62% (13/21) of patients had a complete response. SRI data informed that the randomized portion should proceed with concurrent ibrutinib plus venetoclax, with no ibrutinib lead-in. Ibrutinib plus venetoclax demonstrated promising efficacy; no new safety signals were observed.

*Trial registration*: ClinicalTrials.gov, NCT03112174. Registered 13 April 2017, https://clinicaltrials.gov/ct2/show/NCT03112174.

**Supplementary Information:**

The online version contains supplementary material available at 10.1186/s13045-021-01188-x.

## Introduction

Targeted therapies, such as ibrutinib, have been transformative for patients with relapsed/refractory mantle cell lymphoma (MCL) and have partially replaced traditional chemotherapy and chemoimmunotherapy regimens. However, due to MCL’s aggressive disease course, effective therapies that prolong progression-free survival (PFS) and overall survival (OS) are needed. Ibrutinib, a once-daily Bruton’s tyrosine kinase (BTK) inhibitor, received accelerated approval in the USA for patients with MCL who received ≥ 1 prior therapy [1, 2]. In patients with relapsed/refractory MCL, single-agent ibrutinib induced complete response (CR) rates of 19–28% and overall response rates (ORRs) of 70–77% [3–5], and demonstrated significant improvement in PFS in a phase 3 randomized study versus temsirolimus [4]. With 7.5 years of follow-up, single-agent ibrutinib demonstrated sustained efficacy and no unexpected toxicity for patients with relapsed/refractory MCL [5]. Venetoclax is an oral BCL-2 inhibitor approved in the USA for the treatment of patients with chronic lymphocytic leukemia and previously untreated acute myeloid leukemia [6]. In a phase 1 study of patients with relapsed/refractory MCL, venetoclax yielded a 75% ORR and a 21% CR rate [7]. Ibrutinib and venetoclax have distinct and complementary modes of action and have demonstrated synergistic antitumor activity in preclinical models of MCL, wherein the combination led to increased apoptosis (23%) compared to each agent alone (ibrutinib, 3.8%; venetoclax, 3.0%) [8].

As venetoclax is highly effective at inducing apoptosis, treatment is associated with an increased risk of tumor lysis syndrome (TLS) [9, 10], with label-recommended in-hospital monitoring for TLS development after venetoclax initiation [6]. One strategy to mitigate the risk of developing TLS post-venetoclax initiation is to employ a lead-in with a tumor-debulking agent, such as an anti-CD20 antibody or single-agent ibrutinib [11, 12]. However, given the aggressive nature of MCL, concurrent initiation of ibrutinib and venetoclax may alleviate a potential risk for disease progression (PD) during lead-in.

The phase 3 SYMPATICO study evaluates the once-daily oral combination of ibrutinib plus venetoclax for the treatment of patients with relapsed/refractory MCL. Here, a safety run-in (SRI) was conducted to determine whether concurrent administration of ibrutinib plus venetoclax without lead-in was tolerable. Results from the SRI would inform dosing in the randomized portion of the study.

## Patients and methods

SYMPATICO (PCYC-1143-CA, NCT03112174) is a phase 3 multinational study comprising an open-label SRI cohort and a double-blind randomized period, both conducted in relapsed/refractory patients. An open-label arm in previously untreated patients is currently enrolling (Additional file 1: Figure S1).

In the SRI cohort, enrolled patients (aged ≥ 18 years) had pathologically confirmed MCL and ≥ 1 site of disease ≥ 2.0 cm; 1–5 prior therapies for MCL, including ≥ 1 prior rituximab/anti-CD20-containing regimen; and Eastern Cooperative Oncology Group performance status of 0–2. Patients who had received prior BTK or BCL-2 inhibitors were excluded.

Initiating on day 1, patients received concurrent oral ibrutinib 560 mg once daily and venetoclax starting from 20 mg once daily and ramped up over 5 weeks to a target dose of 400 mg (Additional file 1: Figure S1) [6]. Patients received ibrutinib plus venetoclax concurrently for 2 years followed by once-daily single-agent ibrutinib until PD, unacceptable toxicity, or withdrawal of consent. The study was conducted according to the principles of the Declaration of Helsinki and the Good Clinical Practice guidelines from the International Conference on Harmonization. The protocol was approved by institutional review boards or independent ethics committees at participating institutions, and all patients provided written informed consent.

The primary endpoint of the SRI cohort was the occurrence of TLS and dose-limiting toxicity (DLT) events. A study algorithm was developed to determine the threshold for TLS events and DLTs required to proceed with or without an ibrutinib lead-in (Additional file 1: Figure S2). TLS-risk categories were based on tumor burden and adapted from the US prescribing information for venetoclax [6]. Patients with high tumor burden (≥ 1 lesion > 10 cm or ≥ 1 lesion > 5 cm with circulating lymphocytes > 25,000 cells/mm^3^) and/or creatinine clearance < 60 mL/min at baseline were considered at increased risk for TLS and received TLS prophylaxis and monitoring. Patients not meeting these criteria were considered at low risk for TLS.

Laboratory TLS was assessed per Howard criteria [13]. Clinical TLS was assessed per Howard criteria [13] with modifications (i.e., a diagnosis of clinical TLS required increases in serum creatinine > 1.0 mg/dL from pretreatment baseline); see Additional file 1: Methods for details. DLT was defined as any grade ≥ 3 non-TLS adverse event (AE) at least possibly related to ibrutinib and/or venetoclax occurring during the 5-week ramp-up, which ended on day 7 of venetoclax 400 mg; see Additional file 1: Methods for details.

Secondary endpoints included ORR (i.e., CR, as assessed by positron emission tomography, and partial response per the 2014 Lugano criteria) [14], PFS, duration of response, and safety. Minimal residual disease (MRD) was assessed in bone marrow and peripheral blood by flow cytometry with a detection limit of 5 × 10^−4^.

Requests for access to individual participant data from clinical studies conducted by Pharmacyclics LLC, an AbbVie Company, can be submitted through Yale Open Data Access Project site (http://yoda.yale.edu).

## Results

The SRI enrolled 21 patients (Table 1). Six patients (29%) were considered at low risk for TLS and 15 patients (71%) at increased risk for TLS. All patients had confirmed PD and ≥ 1 lesion > 2 cm at baseline; 11 patients (52%) had baseline detectable MRD in peripheral blood or bone marrow. For patients with available *TP53* mutation data, 5/13 (38%) had mutated *TP53*. Median follow-up was 31 months (range, 1.5+ to 40.2) (Additional file 1: Table S1).

**Table 1:** Baseline demographic and disease characteristics

	Patients at Low Risk for TLS *n* = 6	Patients at Increased Risk for TLS *n* = 15^a^	All Patients *N* = 21
Median age, years (range)	62 (54–67)	70 (53–84)	68 (53–84)
Age category, years, *n* (%)			
< 60	2 (33)	1 (7)	3 (14)
60–69	4 (67)	5 (33)	9 (43)
≥ 70	0	9 (60)	9 (43)
Male, *n* (%)	4 (67)	9 (60)	13 (62)
Median longest diameter of largest lesion, cm (range)	3 (2–5)	8 (2–15)	4 (2–15)
Median circulating lymphocytes, 10^9^/L (range)	1.7 (0.1–3.8)	1.1 (0.4–83.9)	1.2 (0.1–83.9)
Median creatinine clearance, mL/min (range)	108 (72–140)	57 (36–94)	70 (36–140)
Median number of prior therapies, *n* (range)	2 (1–2)	2 (1–4)	2 (1–4)

*TLS* tumor lysis syndrome

^a^Nine (60%) patients had ≥ 1 lesion > 10 cm and 9 (60%) patients had baseline creatinine clearance < 60 mL/min

### Safety

The median treatment duration was 20.1 months (range, < 1–38 months). There were no clinical TLS events. One 74-year-old female patient at increased risk for TLS based on 1 lesion > 10 cm and baseline creatinine clearance < 60 mL/min had onset of a laboratory TLS on Day 2 of treatment. The patient had a medical history of Grade 1 hypertension, and Grade 2 atrial fibrillation, cardiac failure, chronic renal insufficiency, dyspnea, and edema of lower extremities; baseline bone marrow lymphoma involvement was 10%, and baseline white blood cell count was 111.9 × 10^9^/L. The laboratory TLS event lasted 5 days, during which the patient was able to remain on ibrutinib (560 mg) while holding venetoclax (20 mg). For this patient, potassium and calcium remained within the normal range (maximum potassium 4.6 mmol/L, minimum calcium 1.89 mmol/L), while levels were outside the normal range for phosphorous (maximum 2.84 mmol/L), uric acid (maximum 667 μmol/L), and creatinine (maximum 137 μmol/L) during the five-day period. On Day 7, the patient resumed venetoclax (20 mg) (Additional file 1: Figure S3); the white blood cell counts gradually decreased to 5.8 × 10^9^/L on Day 22, and ramp-up to full dose was achieved, with a best response of PR. DLTs occurred in three patients (14%): grade 4 neutropenia lasting > 7 days (*n* = 1), grade 4 infection (*n* = 1), and grade 3 atrial fibrillation plus grade 3 hypotension (*n* = 1).

**Fig. 1:**
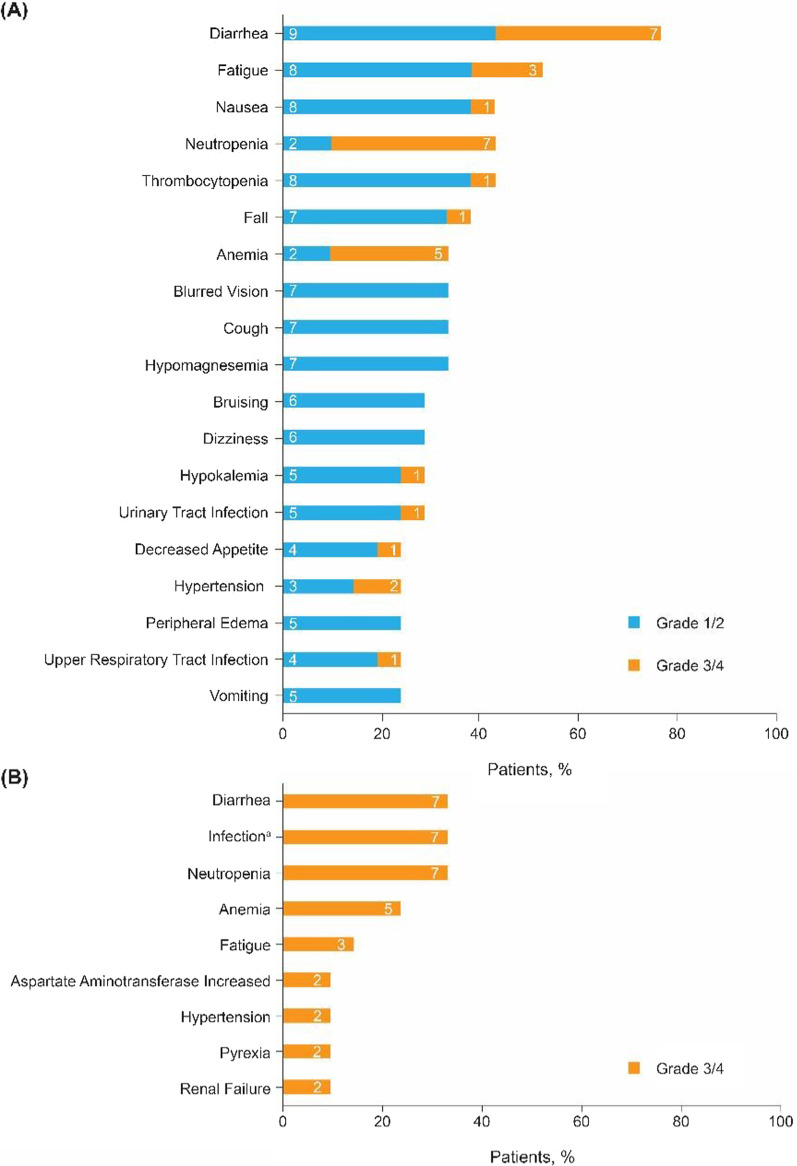
Most common adverse events by grade. **A** Any-grade treatment-emergent adverse events occurring in > 20% of all patients. **B** Grade 3/4 adverse events occurring in > 5% of all patients. Patient numbers are shown within the bars. ^a^AEs of infection were bronchitis (*n* = 1), candida infection (*n* = 1), cellulitis (*n* = 1), fungal abscess central nervous system (*n* = 1, recovered), infection (not specified, *n* = 1), pneumonia (*n* = 2), sepsis (*n* = 1), staphylococcal bacteremia (*n* = 1), upper respiratory tract infection (*n* = 1), and urinary tract infection (*n* = 1)

Most AEs were low grade (grade 1/2) (Fig. 1A). Grade ≥ 3 infections occurred in eight patients (38%). Grade ≥ 3 diarrhea or neutropenia occurred in seven patients each (33%) (Fig. 1B). Grade ≥ 3 atrial fibrillation and hemorrhage occurred in one patient each. Five patients (24%) discontinued both study drugs due to AEs (Additional file 1: Table S2). Four patients (19%) discontinued treatment due to PD.

### Efficacy

**Fig. 2:**
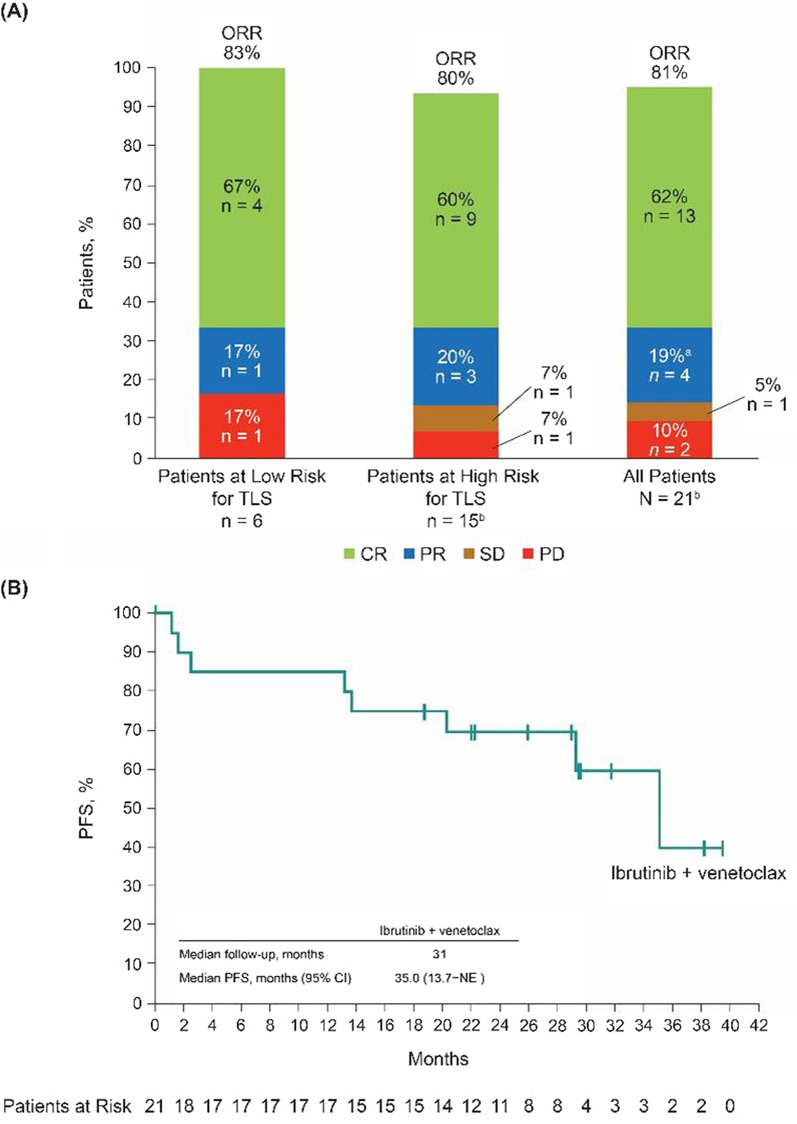
Investigator-assessed efficacy outcomes. **A** Overall response by TLS-risk group. **B** PFS by Kaplan–Meier estimates. Tick marks indicate patients with censored data. Abbreviations: CI, confidence interval; CR, complete response; CT, computed tomography; NE, not estimable; ORR, overall response rate; PD, progressive disease; PFS, progression-free survival; PR, partial response; SD, stable disease; TLS, tumor lysis syndrome*.* ^a^Two patients with a CR by CT scan are missing confirmatory bone marrow examinations and therefore are considered to have PR. ^b^One patient was not evaluable

For all patients, ORR was 81% (95% CI, 58–95%); rates were similar regardless of TLS risk (Fig. 2A). Thirteen patients achieved a CR (62%; 95% CI, 38–82%); all 11 patients with detectable MRD at baseline achieved undetectable MRD. Median duration of response was 32.3 months (95% CI, 26.5–not estimable [NE]). Median PFS was 35.0 months (95% CI, 13.7–NE) (Fig. 2B); the 30-month PFS estimate was 60% (95% CI: 31–80%). Median OS was also 35.0 months (95% CI, 20.7–NE) (Additional file 1: Figure S4).

## Discussion

Results from the SRI of the SYMPATICO study demonstrate that the combination of ibrutinib plus venetoclax is well tolerated and can be safely administered in patients with relapsed/refractory MCL, without the need for an ibrutinib lead-in. To our knowledge, this is the first time that concurrent administration of this treatment combination has been investigated. Notably, a majority of patients enrolled in the SRI cohort were in the increased-risk category for TLS based on tumor burden. In patients with chronic lymphocytic leukemia who are in the high-risk category for TLS, three cycles of a single-agent ibrutinib lead-in have been shown to effectively debulk tumor burden prior to administration of combined ibrutinib plus venetoclax, with 94% of patients shifting from the high category at baseline to medium or low categories after lead-in [15]. In the single-arm phase 2 AIM study, which investigated the treatment of relapsed/refractory MCL with ibrutinib plus venetoclax, a 4-week lead-in period with ibrutinib was utilized, and the TLS-risk category was reduced from high to low in three of seven patients [16]. However, in the same study, two patients never received venetoclax due to rapid PD during the ibrutinib lead-in period in one patient and fatal infection in the other. Given the aggressive nature of the disease, concurrent initiation of the combination is desirable.

In the current study, with the concurrent administration of ibrutinib plus venetoclax, one single instance of laboratory TLS occurred, which resolved in 5 days, and there were no cases of clinical TLS. During the SRI, DLTs were observed in three patients (neutropenia, infection, and atrial fibrillation plus hypotension); however, the DLT incidence did not exceed the prespecified threshold to necessitate an ibrutinib lead-in. Additionally, the safety profile of ibrutinib plus venetoclax was consistent with the known AEs for the individual agents, with no added toxicities. Therefore, based on these data and per study protocol algorithm, it was concluded that the double-blind randomized SYMPATICO cohort for relapsed/refractory MCL would proceed with concurrent initiation of ibrutinib and venetoclax treatment.

Notably, efficacy outcomes in the SYMPATICO SRI were similar to those in the phase 2 AIM study, despite having shorter follow-up (31 vs. 37.5 months) and without an ibrutinib lead-in. In the current study, the CR rate was 62%, and AIM reported a CR rate of 71%, while the median PFS was slightly longer in the SYMPATICO SRI cohort versus AIM (35 vs. 29 months, respectively) [17]. This novel combination appears to be more effective than previously reported single-agent treatment with either drug in relapsed/refractory MCL, as patients treated with single-agent ibrutinib reached CR rates of 19–28% [3–5] and a median PFS of 12.5–15.6 months [4, 5] whereas venetoclax monotherapy induced CR rates of 21% and a median PFS of 14 months [7]. In addition, these preliminary efficacy data from the SRI cohort represent an advancement in either CR rate and/or sustained PFS compared to recent treatment options for patients with relapsed/refractory MCL, including the BTK inhibitors zanubrutinib (68.6% CR rate; 22.1 months median PFS) [18] and acalabrutinib (43% CR rate; 20 months median PFS) [19] or KTE-X19 (anti-CD19 chimeric antigen receptor T-cell therapy; 67% CR rate; median PFS not reached at 12.3 months of follow-up) [20].

In conclusion, SYMPATICO SRI results demonstrate that ibrutinib plus venetoclax was well tolerated without an ibrutinib lead-in. This combination represents a once-daily, all-oral chemotherapy-free regimen for patients with relapsed/refractory MCL. The randomized portion of the SYMPATICO study is evaluating ibrutinib plus venetoclax versus ibrutinib plus placebo in patients with relapsed/refractory MCL. A single-arm, open-label cohort in previously untreated patients with MCL, including those with *TP53* mutations, is also ongoing.

## Supplementary Information

**Additional file 1.** **SUPPLEMENTARY INFORMATION: Supplemental Methods; Table S1.** Patient Disposition; **Table S2.** Safety Summary; **Figure S1.** SYMPATICO study schemas; **Figure S2.** Schema to determine randomized phase 3 dosing; **Figure S3.** Metabolic laboratory values and abnormalities in one patient with laboratory TLS; **Supplementary Figure S4.** Overall survival.

## Data Availability

Requests for access to individual participant data from clinical studies conducted by Pharmacyclics LLC, an AbbVie Company, can be submitted through Yale Open Data Access Project site (http://yoda.yale.edu).

## Abbreviations

AE: adverse event
BTK: Bruton’s tyrosine kinase
CI: confidence interval
CR: complete response
CT: computed tomography
DLT: dose-limiting toxicity
ECOG: Eastern Cooperative Oncology Group
HR: hazard ratio
MCL: mantle cell lymphoma
MRD: minimal residual disease
NE: not estimable
ORR: overall response rate
OS: overall survival
PD: progressive disease
PET: positron emission tomography
PFS: progression-free survival
R/R: relapsed/refractory
SRI: safety run-in
TLS: tumor lysis syndrome
