# Supplementary material for: Concurrent ibrutinib plus venetoclax in relapsed/refractory mantle cell lymphoma: the safety run-in of the phase 3 SYMPATICO study
Source: J Hematol Oncol. 2021 Oct 30;14:179. doi: 10.1186/s13045-021-01188-x (PMC8556975; doi:10.1186/s13045-021-01188-x)

**Additional file 1**

**Supplementary Methods**

Tumor Lysis Syndrome (TLS) Events

For the SYMPATICO safety run-in (SRI) cohort, TLS events were defined as follows:

- Clinical TLS: any event that meets Howard criteria with the following exceptions:
  - For the purpose of TLS assessment during the SRI, only those increases in serum creatinine >1.0 mg/dL from pretreatment baseline will be considered clinical TLS.
  - In patients with renal dysfunction at baseline (creatinine clearance <60 mL/min), clinical TLS is defined as the presence of laboratory TLS plus either seizures, cardiac dysrhythmia, or death.
- Laboratory TLS: any event that meets Howard criteria for laboratory TLS that does not resolve within 72 hours despite protocol-required management.

Dose-Limiting Toxicity (DLT)

A DLT was defined as any grade ≥3 non-TLS adverse event at least possibly related to study drug (ibrutinib and/or venetoclax) and occurring during the DLT assessment period with the following clarifications:

- Nonhematologic DLTs:
  - Grade ≥3 nausea, vomiting, or diarrhea uncontrolled despite maximum medical supportive care and persisting >5 days
  - Grade 3 fatigue persisting >7 days
  - Grade 3 infection was not considered a DLT; however, an infection with life-threatening consequences or requiring urgent intervention (grade 4) was considered a DLT
  - Treatment delay of any study drug >7 days for toxicity
- Hematologic DLTs:
  - Grade 3 neutropenia was not considered a DLT; however, grade 4 neutropenia (absolute neutrophil count <500/mm^3^) lasting for >7 days was considered a DLT
  - Grade 3/4 neutropenia complicated by fever ≥38.5°C or infection
  - Grade 4 thrombocytopenia (platelets <25,000/mm^3^) persisting >7 days
  - Grade 3/4 thrombocytopenia associated with grade ≥2 bleeding
  - Grade 3 anemia was not considered a DLT; however, grade 4 anemia was considered a DLT
  - Treatment delay of any study drug >7 days for hematologic toxicity

Computed Tomography (CT) and Positron Emission Tomography (PET) in Determination of Response Status

Pretreatment tumor assessment was performed within 28 days before the first dose of study drug. A CT scan (with contrast unless contraindicated) of the neck, chest, abdomen, and pelvis and any other disease sites and a PET scan were required for the pretreatment tumor assessment. CT scans with contrast were done for tumor assessments on week 13 day 1 (±7 days); every 12 weeks for the remainder of the first year; every 16 weeks during the second and third years; and every 24 weeks thereafter until progressive disease (PD). In patients with positive PET at baseline, PET or PET/CT was mandatory to confirm a complete response (CR). Disease assessments could be repeated as clinically indicated to confirm response or progression. After the clinical cutoff for the primary analysis of progression-free survival (PFS), all patients without PD continued disease assessments according to standard of care until PD.

If independent CT and PET scanners were used, and the patient was receiving both scans on the same day, the PET must be performed prior to the CT with intravenous contrast. If a CT with contrast scan indicated suspected CR, a confirmatory PET scan could be performed within 30 days.

For patients who remained stable with partial response for >6 months with residual lymph node lesions >1.5 cm that remained relatively unchanged, a PET scan could be performed to determine if CR was achieved and minimal residual disease (MRD) testing should be implemented.

MRD Assessment

MRD assessment was performed by flow cytometry at screening for every patient to detect a dominant clone in peripheral blood or bone marrow aspirate. MRD negativity was assessed by flow cytometry in the peripheral blood and in the bone marrow aspirate collected at documented CR. Patients were followed with peripheral blood MRD analyses by flow cytometry every 12 weeks (±7 days) for the remaining first year, every 16 weeks during the second and third years, and every 24 weeks thereafter until PD. MRD negative remission was defined as undetectable MRD as assessed by flow cytometry of bone marrow aspirate and peripheral blood at documented CR and a consecutive peripheral blood sample 12 weeks later from each patient who achieved a CR.

If the MRD assessment was positive in bone marrow at CR, a repeat bone marrow aspirate was required 24 weeks later if the patient remained in CR to evaluate MRD status in the bone marrow. If peripheral blood became positive for MRD, a bone marrow sample was not necessary. MRD-positive relapse was defined as a detectable increase in disease after MRD-negative remission, as assessed by flow cytometry of a peripheral blood or bone marrow aspirate sample.

**Supplementary Table 1. Patient Disposition**

|  | All Patients  N = 21 |
| --- | --- |
| Median time on study, months (range) | 31.1 (1.5+ to 40.2) |
| Median study treatment duration, months (range) | 20.1 (<1–38) |
| Patient status, n (%)  On treatment  Off treatment/in follow-up  Discontinued study  Death  Withdrawal of consent for follow-up | 5 (24)  6 (29)  10 (48)  8 (38)^a^  2 (10) |

^a^Of eight deaths, two were treatment emergent: one death from adverse event of COVID-19 occurred 5 days after last dose of study drug in a patient with complete response; one patient died from a retroperitoneal hemorrhage due to disease progression. + indicates a censored observation.

**Supplementary Table 2. Safety Summary**

|  | **All Patients**  **N = 21** |
| --- | --- |
| **Grade 3/4 AEs of clinical interest, n (%)**  Atrial fibrillation  Hemorrhage  Tumor lysis syndrome | 1 (5)  1 (5)  1 (5) |
| **Patients with AEs leading to discontinuation of both ibrutinib and venetoclax, n (%)**  Atrial fibrillation  Antineutrophil cytoplasmic antibody–associated vasculitis  Fungal abscess central nervous system  Hepatic function abnormal  Liver disorder  Pancytopenia  Pneumonia | 5 (24)  1 (5)^a^  1 (5)  1 (5)  1 (5)^b^  1 (5)^b^  1 (5)  1 (5)^a^ |
| **Patients with AEs leading to discontinuation of ibrutinib only, n (%)** | 0 |
| **Patients with AEs leading to discontinuation of venetoclax only, n (%)**  Abdominal pain  Diarrhea | 1 (5)  1 (5)^c^  1 (5)^c^ |

AE, adverse event. ^a^One patient discontinued both ibrutinib and venetoclax due to atrial fibrillation and pneumonia. ^b^A second patient discontinued both ibrutinib and venetoclax due to hepatic function abnormal and liver disorder. ^c^One patient discontinued venetoclax due to abdominal pain and diarrhea.

**Supplementary Figure 1. SYMPATICO study schemas.** In SYMPATICO, patients will receive oral, once-daily ibrutinib 560 mg plus venetoclax in a 5-week ramp-up to 400 mg. Patients receive ibrutinib plus venetoclax for 2 years; after 2 years, venetoclax is discontinued in all patients, and single-agent ibrutinib continues until disease progression or unacceptable toxicity. ^a^Enrolling sequentially; data are to be presented in the future. ^b^Including approximately 25 patients with a *TP53* mutation. Abbreviations: ECOG PS, Eastern Cooperative Oncology Group performance status; MCL, mantle cell lymphoma; PD, progressive disease; R/R, relapsed/refractory; TLS, tumor lysis syndrome.

**Safety Run-in**

**
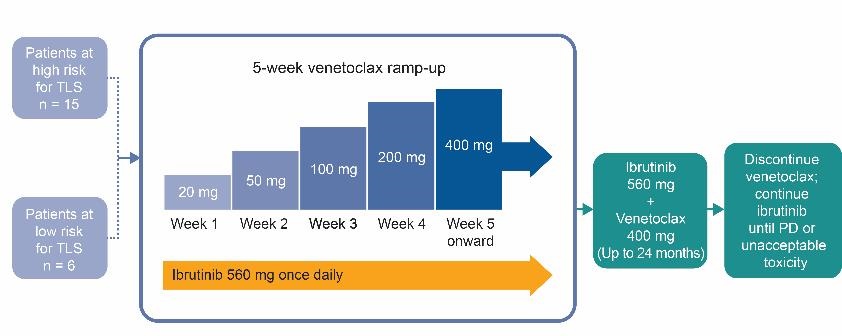
**

**Randomized R/R MCL^a^ and Open-Label Previously Untreated MCL Cohort^a^**

**
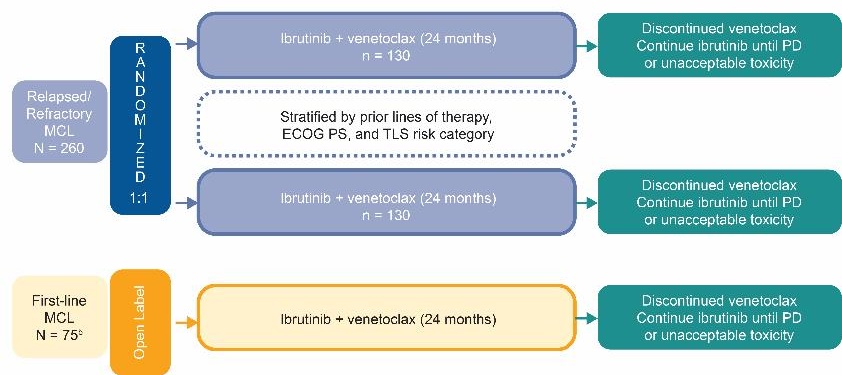
**

**Supplementary Figure 2. Schema to determine randomized phase 3 dosing.** Abbreviations: DLT, dose-limiting toxicity; SRC, scientific review committee; TLS, tumor lysis syndrome.

**
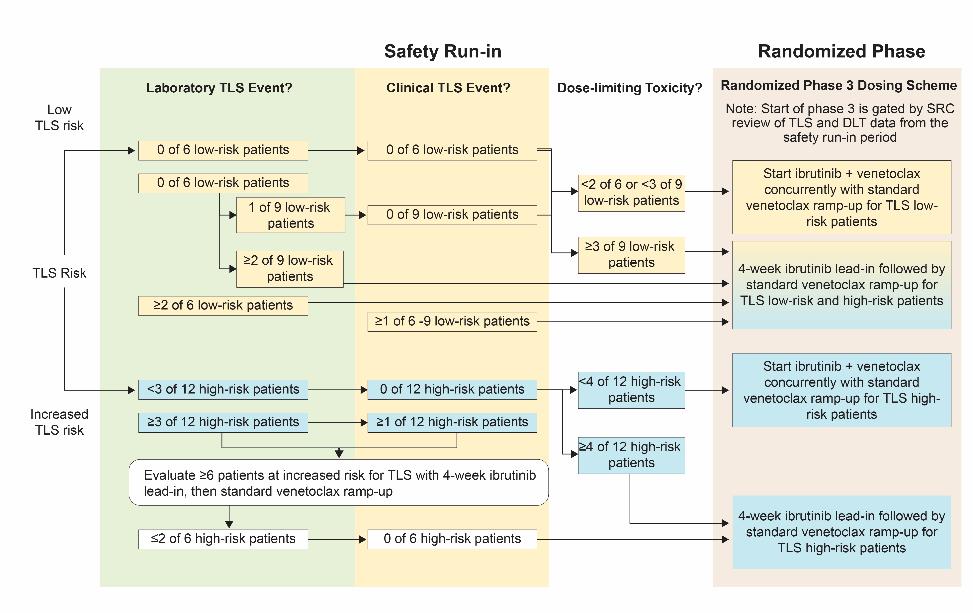
**

**Supplementary Figure 3. Metabolic laboratory values and abnormalities in one patient with laboratory TLS.** ^a^Maximum blood chemistry levels were potassium, 4.6 mmol/L; phosphorous, 2.8 mmol/L; uric acid, 667 μmol/L; and creatinine, 137 μmol/L; minimum level of calcium was 1.9 mmol/L. ^b^Increase in creatinine >26.5 μmol/L from 106 to 137 μmol/L (=31 μmol/L).


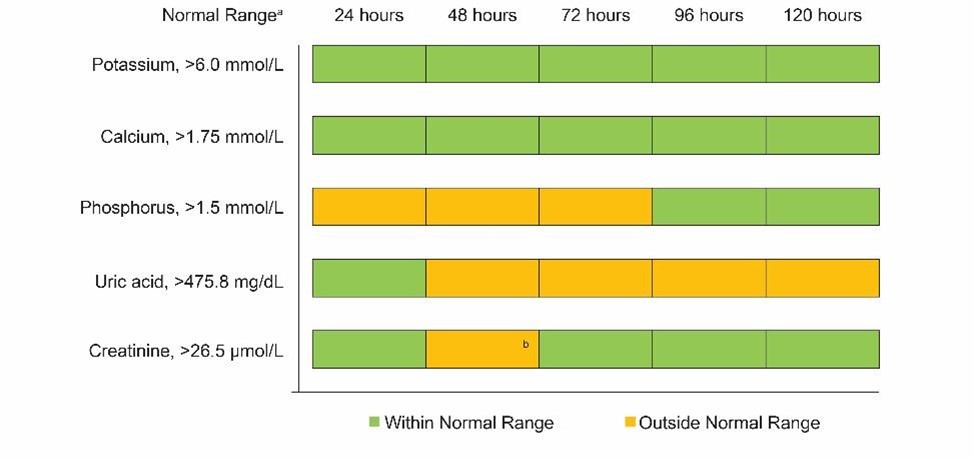


**Supplementary Figure 4. Overall survival.** Tick marks indicate patients with censored data. Abbreviations: CI, confidence interval; NE, not estimable; OS, overall survival*.*


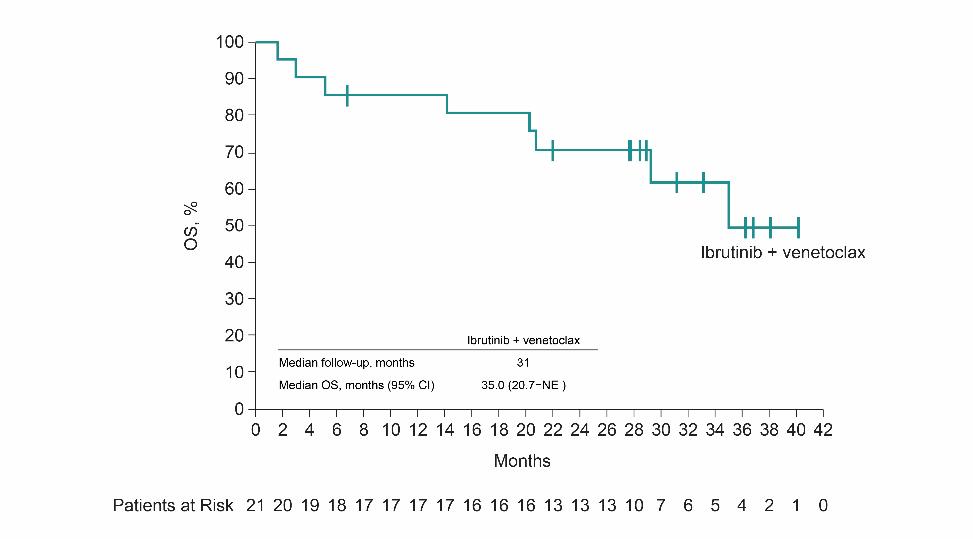

Supplement: Supplementary file 1 — Additional file 1. SUPPLEMENTARY INFORMATION: Supplemental Methods; Table S1. Patient Disposition; Table S2. Safety Summary; Figure S1. SYMPATICO study schemas; Figure S2. Schema to determine randomized phase 3 dosing; Figure S3. Metabolic laboratory values and abnormalities in one patient with laboratory TLS; Supplementary Figure S4. Overall survival. [file 13045_2021_1188_MOESM1_ESM.docx]
